# Perspective of stem cell research & therapy in diabetes

**DOI:** 10.1186/1755-8166-7-S1-I61

**Published:** 2014-01-21

**Authors:** Sarita Gupta

**Affiliations:** 1 Department of Biochemistry, Faculty of Science, The Maharaja Sayajirao University of Baroda, Vadodara, India

## 

In the area of regenerative medicine, researchers have been exploring the potential of Stem Cells in preclinical and clinical studies to improve therapies that can resolve injuries by enhancing endogenous repair, opening a new paradigm in cell therapy.Stem cells may it be embryonic stem cells (ESC), induced pluripotent stem cells (iPSC) or adult stem cells have been used as potential sources for beta cell replacement therapy across the globe. Numerous studies have shown the differentiation potential of ESCs and iPSCs towards producing pancreatic progenitors but after terminal differentiation into insulin producing cells these lacked various important pancreatic markers and have low index of glucose stimulated insulin secretion (GSIS). This along with other ethical conundrums makes it difficult to use ESCs as a source for cell therapy. Adult stem cells provide clinically and ethically accepted option over embryonic or induced pluripotent stem cells, as they can be used as autologous source for cell transplant in treatment of diabetes. Widely used option of cadaveric islet therapy has its own problems due to insufficient amount of islets available for transplantation. Hence, our research is focused on increasing islet mass from various adult stem cells using neutraceutic bioactive compounds isolated from a plant demonstrating efficient anti-diabetic activity and further explored them as potent differentiating agent under *in-vitro* and *in-vivo* condition. *In-vitro* differentiated islets from various adult stem cells sources were assessed at morphological, molecular, immunological and functional level and further evaluated for glucose lowering effect after transplantation in Streptozotocin (STZ) induced diabetic *balb/c* mice.

